# Family-school relationship in the Italian infant schools: not only a matter of cultural diversity

**DOI:** 10.1186/s40064-016-3581-7

**Published:** 2016-10-26

**Authors:** Anna Granata, Ouejdane Mejri, Federica Rizzi

**Affiliations:** 1 Department of Philosophy and Educational Sciences, University of Turin, Via Giuseppe Verdi n. 8, 20124 Turin, Italy; 2 Centre of Excellence Altiero Spinelli, Rome 3 University, Rome, Italy; 3 Catholic University of Milan, Milan, Italy

## Abstract

**Background:**

The family-school relationship is a crucial component in achieving the optimum scholastic experience of pupils. Such a relationship is often described in somewhat reductive binary terms of collaboration/non-collaboration. However, the significant presence of families from different cultural backgrounds in Italy since the 1990s demonstrates how multiple types of rapport with schools can generate effective styles of relationship. Infant schools constitute a privileged location where such dynamics can be investigated. Firstly, because they exhibit the highest percentage of families that have moved to Italy from other countries (33.9%); secondly, because they represent the initial stage when school and family first come into contact, playing an “imprinting role” for all subsequent scholastic phases. Based on in-depth interviews with infant school teachers and parents of pupils coming from different backgrounds, this research investigated different factors that influence family-school relations: (1) interpersonal factors, that include listening skills, emotions and relational styles of parents or teachers; (2) structural factors, that are related to the living conditions of families and to the whole social welfare system in Italy; (3) cultural factors, that bring together values, lifestyles and educational cultures of both parents and teachers.

**Results:**

The idea regarding the inadequate distinction based on a dichotomy between Italian and migrant families seemed to be confirmed: Italian families and migrant families are characterized by many common features as well as by many inner differentiations. The results of this study suggest that the family-school relationship evolves into a communication framework that encompasses both obstacles and resources.

**Conclusions:**

The results of our research suggest that the relationship between parents and teachers in Italian infant schools is influenced by different factors, not only cultural ones. All of these factors are related to both parents and teachers, and they should be taken into account when investigating this topic.

## Background

The evolution of migration in Italy developed exponentially during the eighties when the families of migrants (i.e. wives and children) were able to join their husbands/fathers, who constituted the core migrant population during that period. Rules concerning migration were promptly accompanied by institutional considerations and regulations in relation to the increasing presence of children of immigrants in Italian schools.

Since 1990, Italy has embraced the full integration of everyone in the school and intercultural education as its cultural horizon (Mpi 1990). Compared to other European countries, where migration has a longer history, such as France and the UK, Italy rejected, on the one hand, the assimilation approach and, on the other, the erection and strengthening of inward looking ethnic communities. Over the years the idea of implementing intercultural education was promoted in order to encourage comparison, dialogue and mutual enrichment generated by coexisting differences as set out in regulatory texts (Mpi 2006).

The 2003 Italian school reform highlighted the role of families in their children’s education process. In its article 1 it refers to the relationship between school and family, involving the latter in the definition of the education portfolio and customized educational plans (Mpi 2003). Families (immigrants and non-immigrants) are in this way encouraged to assume an active role during their children’s educational path as part of a collaboration framework with schools. This law expressed a shift of cultural emphasis: from a model where schools were offering the same training to all pupils to a model where schools shape their educational “offer” on the basis of individual needs. In this way Italian schools seek to adapt to their students, whose “specificities” have to be considered from the outset. This reform reinforced an intercultural trend which considers differences as a factual part of the education system. Indeed, the Ministerial Directive of 2007, entitled “The Italian approach to an intercultural school and to the integration of immigrant pupils”, indicates that teaching in an intercultural context means assuming diversity as a paradigm of the school itself, a privileged opportunity to embrace all differences (Miur 2007).

However, it is worth underlining that the educational model in Italy is characterized by the autonomy enjoyed by individual schools in their choices and introduction of innovative practices and actions. This aspect is decisive in the resulting outcomes which intercultural approaches have produced in some cases registering a quantum leap in the realisation of such an approach, in others failing to do so.

The 2007 national guidelines for the integration of immigrant pupils make it clear that relationships with migrant families are part of the actions for such integration. More specifically, following the arrival of a migrant family in Italy, a *welcome* phase aims to establish an educational pact with the migrant family, considered as a fully educational partner, in order to construct the basis for positive collaboration between the two educational spaces (Miur 2007). A further description of the styles of relationship has been developed in these guidelines, specifying that the active and co-responsible participation of migrant families in the initiatives and activities of the school, and knowledge sharing relative to the educational project, are part of an *alliance* that enhances educational specificities (Miur 2007).

Within this context, our research focused on the family-school relationship and aimed to characterize the obstacles faced and opportunities offered to families in developing a positive relationship with schools.

We decided to explore the relational factors determined by family-school relationship dynamics within nursery schools, since they constitute a privileged location where such dynamics can be investigated. Nursery schools in Italy, referred to since 2003 as “Infant Schools”, offer early childhood education to children between the ages of three and five, preparing them for subsequent compulsory education at primary school level. Infant schools in Italy, in their different institutional forms (i.e. public, private, religious, associative etc.), are regulated by ministerial norms which consider them as part of the national educational system.

These establishments host the highest number of migrant families in the whole educational system and, more importantly, they represent the initial stage when school and family first come into contact, playing an “imprinting” role relative to all subsequent scholastic phases.

In exploring the relationship between the two educational spheres constituted by infant schools and families, we tried to assess those communication obstacles which may generate problems during the implementation of an efficient relationship between the two parties, highlighting where possible all relevant emerging resources.

## Methods

The methodology of this research was qualitative. The study was conducted between December 2014 and July 2015 in three infant schools in Milan (Italy). In order to guarantee as much as possible the socio-economic heterogeneity of the sample, although its small size, the three infant schools were selected in three different areas of the city, each one with a different percentage of immigrant families, as follows:

- school 1: central district of the city, medium–high class, presence of professional parents and low percentage of children of immigrants;
- school 2: heterogeneous district in the south of the city, with medium–high class families and families from working class housing, mainly immigrant;
- school 3: suburban districts in the south-east of the city, with a prevalence of families from working class housing, lower class, mainly immigrant.

A convenience sample of ten participants, five teachers and five parents, was selected. The five teachers enrolled in the study were contacted by an email circulated among the participants of a previous training course for teachers about intercultural education. The first five teachers that answered the email were considered for interviews. The five parents enrolled in the study were contacted directly by each teacher (one each). Inclusion criteria for parents were: living in Italy from at least 5 years and a sufficient knowledge of the Italian language. All teachers were female and the mean length of their experience in the school system was 17.2 years (CI: 5–24). The parents (4 female and 1 male, mean age 29.4 years, CI: 26–34) were living in Italy from an average of 10.2 years (CI: 5–15), and came from five different countries (Italy, Tunisia, Sri Lanka, Morocco, United States of America). Their educational and socio-economic backgrounds were heterogeneous. All participants signed an informed consent form prior to the study.

We decided to interview both teachers and parents to explore affinities and dissonances relative to elements perceived as resources and obstacles to relationship by both parties.

The data collection was carried out using the method of “in-depth interviews” and in written form via “personal stories” (Audet 2008; Gervais and Desgagné 2003). All the interviews, one with each participant (both teachers and parents), were conducted by the same interviewer and lasted 2 h.

Interviews were semi-structured and started with a brief description of the research. The questions of the interview aimed to investigate different domains, as follows:

- For teachers: school and school class characteristics (how many students, socio-economic background, presence of resources or limiting factors due to diversities among children), relationship with children’s families (presence of conflicts and positive aspects, examples of eventual positive changes in the relationship with parents), suggestions to improve the relationship with the families.
- For parents: family and personal characteristics (members, age, who usually care for children, eventual presence of other educators they’re in contact with), relationship with the school (who are the family members that usually interact with the school, in which occasions, with whom), quality of the relationship (how they consider the relationship with the school, are they satisfied about it, eventual episodes of conflict or good collaboration), suggestions to improve the relationship with the school.

At the end of each interview participants were invited to think about the content of the interview and eventually to write about a positive or a negative episode concerning their relationship with teachers and parents (for parents and teachers respectively).

The participants were encouraged to talk about their relationship experiences with their interlocutors (teachers for parents and vice versa), referring to both positive and negative episodes.

This method encourages self-reflection and makes it possible to disclose, without rhetoric or abstractions, difficulties and strengths in the relationship among teachers and parents.

In interpreting the data collected, emphasis was placed on the facts reported by parents and teachers, but also on the meanings they attributed to these, highlighting both the pragmatic and hermeneutic aspects of intercultural reflection (Abdallah-Pretceille 2003, 2007; Eisenhart 2001). Information about the factors influencing the family-school relationship was extracted and synthetized into Tables 1 and 2. The analysis was carried out by an independent operator (the interviewer didn’t take part in the analysis).

**Table 1:** Factors influencing an effective family-school relationship from the teachers’ point of view

	Teachers’ point of view	
	Obstacles	Resource elements
Interpersonal factors	Stereotypes and prejudices relative to some families of low social class and foreign origin	A relationship of trust towards teachers and school
	Distrust on the part of parents	
	Parents’ poor knowledge of the Italian language	
Structural factors	Unvailability of an extended family network	New communication technologies
	Parents’ work schedules incompatible with those of the school	
Cultural factors	Distrust towards of teachers	Valorising parents’ culture of origin
	Poor understanding of teachers’ professional role	
	Excessive respect for teachers’ and school’s authority

**Table 2:** Factors influencing an effective family-school relation from the parents’ point of view

	Parents’ point of view	
	Obstacles	Resource elements
Interpersonal factors	Stereotypes and prejudices relative to some families of low social class and foreign origin	Trust towards teachers, including when difficult situations arise
	School vocabulary	
	Gender issues	
Structural factors	Economic contribution to the life of the school (not all families can afford it)	New communication technologies
	Parents’ work schedules incompatible with those of the school	
Cultural factors	Aims of the school not always clear	Valorising parents’ culture of origin

## Results

In our analysis of results we distinguished between obstacle and resource factors in the family-school relationship, exploring both points of view, that of teachers as well as that of parents. In the discussion we reveal the numerous features held in common by the two “voices”, indicating factors perceived as significant by both parties.

### Point of view of teachers

The teachers involved in the research all work in the city of Milan, with a population of two million inhabitants, where children of immigrants have been increasingly present since the 1990s. The presence of immigrant families varies considerably from district to district, with very marked ethnic and social class segregation dynamics.

The teachers we interviewed were exclusively women, with various levels of experience in the school (from those who had just taken their degrees to professionals with 20 years of experience, who have seen first-hand the socio-demographic changes in Italian schools). Female teachers in Italy are very much in the majority and almost totally dominate education services in infant schools.

Interviews with teachers and their “personal stories” revealed various obstacle and resource factors in the relationship with the families of the children they teach.

First, there are some obstacles linked to *interpersonal factors*, such as the stereotypes and prejudices of teachers relative to some families, often of low social class and immigrant. Such stereotypes and prejudices are generated by diffidence towards others, with direct effects on the children of such families:

In my class, immigrant parents are considered “inferior” with respect to Italian parents by the other teachers. […] The older colleagues in particular are afraid of immigrants; it’s not racism, nor malice, it’s fear. […] Phrases like “they think they are owed everything”, “they never attend parent-teacher evenings”, “they make no attempt to understand”, “they never show up”, “despite everything we do for their child”, “they always make their child wear the same clothes” are generated by their prejudices and have an effect on the children, who are viewed as direct products of their parents. The majority of parents affected by these kinds of prejudices are immigrants or those belonging to a low socio-economic class (Teacher, school 2).

On the contrary, an attitude of trust on the part of parents appears to teachers to be the best relational resource in building solid and positive relationships. For one of the teachers involved, this trust was already established in the preliminary meeting with the families: “On that very first day I ask parents for a “blank cheque”, if they decide to entrust their children to me. Otherwise we cannot collaborate. Trust must almost be demanded from parents. If we have a relationship of trust the child will succeed in positively fitting in at school.” (Teacher, school 1).

Another relational factor that sometimes obstructs family-school relations is, according to teachers, the poor knowledge of Italian on the part of some families who have only recently immigrated to Italy, as has already been noted in other research work. “Many immigrant parents do not speak Italian well; there is therefore a communication problem which has not been taken into account by the schools. A course in Italian for Arab mothers would certainly facilitate their integration…” (Teacher, school 3). This linguistic barrier has emotional implications for the relationship between parents and teachers, in a context that makes little or no attempt to encourage multilingualism or communication exchange by means of various linguistic registers.

*Structural factors* also positively and negatively influence family-school relations. These include, as an obstacle element, the absence of a family network extended over the territory. This is especially problematic in a welfare system like that in Italy, based on informal extended family support to help take care of children, rather than on care services offered to families: “parents of immigrant children never turn up for parent-teacher meetings, almost certainly because they have no support from an extended family network, grandparents, uncles, aunts etc.. […] Many parents do not come to the meetings because they have no one they can trust to look after their children…” (Teacher, school 2).

Among structural factors there is also the difficulty experienced in reconciling work hours with school times, an element that is also cited in the narrations provided by parents. A teacher described a specific case: “The father of a child, of Romanian origin, came to a parent-teacher pre-registration meeting in work clothes (bricklayer), apologising for the way he was dressed. […] After that, he never came to any parent-teacher meetings; I think his work times prevented him from taking part” (Teacher, school 3). In the opinion of another teacher, the school should have open times other than those normally offered, like Saturday morning, to facilitate the participation of such parents in the life of the school.

On the other hand, in the family-school relationship, communications are nowadays often carried out using techniques other than traditional “notices” hung up in front of the class, often ignored by the majority of parents. Emails or “whatsapp groups” have become an instrument for communication between families and even sometimes between teachers, to transmit practical information or facilitate reciprocal knowledge, including over long distances. However, teachers do not hide the fact that there are some negative features relative to this type of communication: “not all parents have internet on their mobile phones, so some are excluded from this type of communication” (Teacher, school 2).

Finally, *cultural factors* undoubtedly influence family-school relations both positively and negatively. In our research work we identify as cultural factors those attitudes and behaviours displayed by both Italian and immigrant families, according to an approach where culture is seen as something that native people also display, and not only immigrant families.

This kind of greater awareness appears to be infrequent even among teachers, who identify as cultural behaviour solely that of immigrant parents, as indicated by the story of a teacher:

There is an Egyptian family which is “very Arab”. For example, it focuses much more attention on the boy than on the girl. They find it difficult to adapt to the rules of the school, always arrive late for parent-teacher meetings. […] The mother of an Italian boy always arrives late too, and rarely takes part in parent-teacher meetings. She is a university lecturer… a professional woman! She never has any time… (Teacher, school 1).

This affirmation clearly reveals how in the worldview of this teacher the behaviour of the immigrant family is interpreted through a generic and in some senses stereotypical cultural prism; while the very same kind of behaviour, exhibited by an Italian family, is not attributed to cultural but to merely personal factors, linked to professional standing. In other words, the cultural approach is used to describe the behaviour of “others” but not that of Italians.

We have also noted how among the cultural factors that can hinder family-school relations there is, according to some teachers, the diffidence and poor understanding of their professional role, with a consequent diffidence towards the school as an institution. A feature that seems more widespread among Italian families of medium–high class: “with my colleague we decided to ensure information notes were signed by mothers who were given information in order to avoid any later misunderstandings or disagreements. It often happens that Italian families complain they have not been informed relative to this or that matter…” (Teacher, school 1).

On the other hand, teachers have noted excessive respect shown to them by some immigrant families, coming from social contexts where there is no provision for any active participation of parents in the life of the school:

Immigrant parents completely delegate the education of their children to the scholastic institution and do not intervene in school questions, not least because they fear it will be perceived as a lack of respect for the school’s authority. We have particularly noted that parents from Arab countries often display this type of behaviour (Teacher, school 3).

This latter type of behaviour can be explained by the poor or non-existent opportunities for participation in the life of the school in the social contexts they come from. The scholastic experience of parents represents an essential element in defining their expectations and relationship modalities with their children’s school, including in a different geographic and temporal context.

In some cases an unusual way of relating with the school is described, as in this example provided by another teacher:

The father of a Chinese girl often came to school: every time he was asked to make an economic contribution (for teaching equipment, school trips etc.) he paid immediately, often without even knowing what the money would be specifically used for. Giving money was the most immediate thing, in his opinion, that he could do to take part in the life of the school (Teacher, school 3).

In this case too, it is likely that the parent of Chinese origin acquired this particular way of relating to the school as an institution in his country of origin, and felt that this was the best way to collaborate with the Italian school.

On the other hand, another teacher focused on a specifically cultural factor, when relating an episode: she was speaking to the mother of a three year old boy who originally came from China. The boy was very agitated at school and the teacher asked for explanations from the mother. The mother, who spoke little Italian, told the teacher to “put the boy with his face to the wall”, as a form of punishment, which she felt would be effective. The teacher was not accustomed to this type of method, but decided to adopt the mother’s suggestion, given that it seemed the boy was used to this style of education at home. “After 2 h, I remembered that X was still standing with his face to the wall! I had completely forgotten that I had left him there … I am not accustomed to using these methods. He had remained standing there, ‘as good as gold’, facing the wall” (Teacher, school 2).

Cultural factors can also be seen as resource elements in the family-school relationship; teachers unanimously state the importance of valorising the culture of origin of the parents as a factor that facilitates their relationship. The following are the words of one of the teachers who accurately describes the importance of this factor:

Parents, especially those who are not Italian, want to talk about themselves: often they feel it is important to specify that in their country of origin they had a good education, they obtained their degree, they lived in a beautiful house; they do not want to be viewed solely in the light of their current situation in Italy, but for their whole life experience e.g. an African mother who works in Italy as a cleaning lady but when she lived in her home country she was a teacher (Teacher, school 2). In Table 1, we summarize the obstacle and resource aspects that emerged from the teachers’ accounts.

### Point of view of parents

The parents involved all live in the city of Milan, in districts that vary considerably in terms of their socio-cultural contexts.

The parents we interviewed were all women, with the exception of one father, aged between 30 and 40, with very different socio-economic conditions and an array of professional experiences (from university lecturer to factory worker), of various origins (Italy, Morocco, Tunisia, Sri Lanka, United States), different migratory experiences and periods of stay in Italy (from 5 to 20 years).

Participation in the life of the school, and the consequent decision to accept taking part in an interview on this subject, directly involved far more mothers than fathers.

Interviews with parents brought to light various obstacle and resource factors in their relations with the teachers of their children. The parents involved in the research, like the teachers, described a series of obstacles and resources that influence their relationship with their children’s school.

First, some *interpersonal factors* arose as stereotypes and prejudices, both positive and negative in character. We feel it is interesting to set out one example linked to the Christmas season. One mother told us the following:

Christmas was approaching. The school director called me and asked me to act as a “mediator” with Muslim mothers to reassure them as regards the activities that would be taking place during this festivity, which included a visit to a church. He wanted me to tell the mothers that “their children would not become Christians”, as the manager put it. In reality, the Muslim mothers reacted in a completely different way: they were not angry or irritated, but simply wanted to take part, cooking or preparing things that would be useful for the festivity. The director’s fears proved to be completely unfounded… (Parent, school 2).

As this story makes clear, prejudices, including of a positive character, can be obstacles to relationship if there is no opportunity to overcome them. The subject of religion is one of the most delicate and misunderstood areas in the relationship with immigrant families. The idea that Muslim families could feel offended or awkward about festivities linked to the Catholic faith is often disproved by the facts and the openness of Muslim families, for whom religion is important, including when it does not involve their own faith.

Prejudices against families are not limited, however, only to parents of other origins or religions. Italian families too are considered to display certain typical features, e.g. they are “too involved” in the life of their children’s school:

Teachers often negatively judge the families: they feel they are too intrusive in their children’s education and training. Teachers are very “protective” about their didactic projects and work, do not accept suggestions from parents, even if the latter have specific skills in certain areas (Parent, school 1).

The subject of excessive interference on the part of Italian families in the life of the school is linked to the question of different roles carried out by the family and the school, roles that sometimes appear to be managed in an ambiguous manner. As an immigrant mother explained, “teachers should not be vice-mothers” (Parent, school 1).

On the other hand, a factor that facilitates family-school relations, in the words of parents, appears to be once again trust, including when difficult situations arise, as in a very effective example described by the mother of a young boy:

Last year, two children ran away from school during an activity. They wandered around the city, crossing a very busy road. The subject was tackled in class: the school admitted they bore responsibility, the teachers that they were to blame. The parents did not get excessively angry with the teacher responsible; there was a good level of mutual understanding between the parents and the school institution. It was an excellent opportunity for collaboration, even though it was a delicate situation (Parent, school 2).

An obstacle factor, in the opinion of immigrant families, in the family-school relationship, is represented by the language used by teachers. Not linguistic difficulty, linked to a poor knowledge of Italian, but the language used by schools that have codes and vocabulary (jargon) that are not always easy to understand. As already emphasised by other research work, words like “equivalence”, “portfolio”, “primary school”, “training credits” made even worse by the use of acronyms like “pof”, “nai” or “invalsi,” which are certainly not easy for families to communicate the idea of a system that is difficult to interpret, especially for families that have come from other countries.

One father interviewed identified the question of gender as a source of difficulty: relations with the school are mostly undertaken by mothers and the teachers are for the most part women. This fact is closely connected to the question of school hours, often incompatible with work schedules: “I work from 5.30 to 18.00, there is no way I can ever accompany my daughter to school, or pick her up” (Parent, school 3).

Interpersonal facts are accompanied by *structural factors* which influence family-school relations. These include, and were named by many parents, socio-economic factors. In the current historical period schools ask parents to make an economic contribution to the life of the school, but not all families can afford to do so: “The discussions among parents almost always focus on questions concerning money, the economic contributions requested relative to activities. Not all families can afford to pay these…” (Parent, school 2). Among structural factors, on the other hand, that facilitate the family-school relationship there is again mentioned by parents the experimentation of forms of communication that differ from traditional ones, like “whatsapp groups”.

With respect to *cultural factors*, it became clear that parents like the valorisation of their cultural origin as an element that facilitates reciprocal trust between teachers and immigrant parents. One father stated that he would be very happy to talk about Morocco in his daughter’s school: “I would do it for her, for the other children” (Parent, school 3).

But there is another element that seems very interesting relative to intercultural considerations. Various parents make it clear that the aims of the school and the expectations of teachers are not always clear, a feature already identified as typically voiced by immigrant families and also voiced by Italian families in our research.

The perception of the role of the school in the lives of children depends to a considerable extent on the experience parents acquired in their countries of origin, where frontal didactics, discipline, affective distance, rigid imposition of rules and the authority of teachers are still very much a daily reality.

This factor is shared by both Italian and immigrant parents: what counts is their own scholastic experience which they seek, as a point of reference, in their children’s schools.

It is vital that these expectations receive an answer:

Often I don’t understand what teachers want from me as a parent, it’s difficult to understand what kind of contribution they want from the families in the school… School in Tunisia is a frontal school, families are absolutely not involved in scholastic and education dynamics, it is the teacher who, with total autonomy, decides on the scholastic programs and methods. We don’t understand what they want from us! (Parent, school 2).

This declaration makes it clear that schools are failing to communicate their aims and expectations in their relations with parents. A factor that certainly does not help parents from other cultural contexts to consolidate their role within their children’s school, even when they are fully prepared to collaborate. Table 2 provides a summary of the obstacle and resource aspects that emerged from our interviews with parents.

## Discussion

The subject of the family-school relationship in Italy has been extensively explored by educational literature, but most of the studies concentrated on Italian families or immigrant families distinguishing specific aspects of these two populations (Adair and Tobin 2008; Tobin et al. 2013; Bove et al. 2010; Catarsi 2008; Dusi 2012; Mantovani 2006; Silva 2006). Although this approach was certainly valid in exposing the specificity of these experiences, today the situation appears decidedly more complex. A homogenous representation of immigrant families has become less effective and justified—distinguished internally by place of origin, migration times, religious, cultural and other more personal aspects, as was also found in some studies concerning the same research field in other countries (Hadley and De Gioia 2008; Peña 2000).

Within the Italian social context research relative to family-school relations in this specifically intercultural context is still limited (Maggioni and Vincenti 2007; Santerini 2010; Zoletto 2007). The present research contributes to this theme, one we can refer to as “intercultural” precisely because it tries to intercept both the point of view of teachers and that of families, including both Italian and immigrant families.

The chosen infant schools were selected because they represent a privileged place in which to investigate the family-school relationship from an intercultural point of view, as reported in some studies of foreign experts interested in the state of education services and policies in Italy (New et al. 2000; Tobin et al. 2013).

Our survey aimed to describe the family-school relationship from the point of view of the practices and imagination of parents and teachers, following the “alterity paradigm” of Martine Abdallah-Pretceille (Abdallah-Pretceille 2003). This paradigm demonstrates that any relationship with another person occurs in a heterogeneous context, never completely egalitarian, in which different factors intervene (interpersonal, socio-economic, cultural). The individual is not considered just as a product but above all as a producer of his or her culture. This is the reason why we choose to concentrate our focus on both Italian and immigrant families avoiding the traps of ‘culturalization’ (considering culture as the only factor in human relationships) and indifference (avoiding consider culture as a factor in human relationships) (Ogay and Edelmann 2016).

The results of this study suggest that the family-school relationship evolves into a communication framework that encompasses both obstacles and resources. This framework may be described by the following set of factors:

- Interpersonal factors: these are factors that condition direct communication between teachers and families. This communication includes listening skills, exchange and sharing of information, as well as the ability to collaborate in specific tasks. Interpersonal factors include emotions, attitudes and relational styles of parents or teachers, which can affect the family-school relationship;
- Structural factors: these are factors related to the living conditions of families and also to the whole social welfare system. Firstly, they indicate the socio-economic situation of families and the different organizational conditions (e.g., working hours, availability of an extended family network, access to new technologies, etc.). Secondly, these factors relate to the presence or absence of services provided by the national welfare system and the degree of access to these services, elements that influence the family-school relationship;
- Cultural factors: these are factors that bring together values, visions and imagination, lifestyles and educational cultures, linked in some way to the cultural backgrounds of both individuals and groups. These are factors that characterize both families and schools and clearly influence the relationship between them.

The idea regarding the inadequate distinction based on a dichotomy between Italian and migrant families seemed to be confirmed: it can be seen that Italian families and migrant families are characterized by many common features as well as by many inner differentiations. A culturist vision has characterized intercultural educational research for a long time and risks considering cultural factors only in relation to migrant family experience, leading one to imagine a sort of “immigration culture” that is actually non-existent when we consider the degree of pluralism present in the origins and life phases of such families (Abdallah-Pretceille 2007). In the meantime, the “stereotype”, sometimes conveyed even in intercultural educational studies, where immigrant families have more problematic relationships with schools than Italian families is called into question by the obstacles and resources which teachers have identified in the behaviours of both Italian and immigrant families.

A further issue which may be particularly interesting for intercultural debate arises from both teachers and parents, Italians and immigrants, concerning difficulties in understanding the aims of the school and its operators. This may be related to comprehension difficulties on the part of families, but it also seems to be due to the limitations of schools in clarifying their own objectives and making them effective, including their relationships with the children’s families.

Similar results (i.e. lack of trust, language difficulties in communication, school experiences of parents in communicating with school, the need for school flexibility in accommodating working parents’ shift work) were also reported in studies on the same topic conducted in other countries (Audet 2008; Crozier 2014; Dusi 2012; Hohl and Normand 2000).

The limiting factors of this study included mainly the small sample size. With a bigger sample the impact of certain factors (i.e. gender, economic) on family-school relationship might have been better understood, and any further elements concerning interpersonal, structural and cultural factors may have been reported.

## Conclusions

Considering this study in an intercultural context, it could be stated that a cross-fertilization of Italian and immigrant family experiences with schools is an essential option, one that should be pursued to overcome prejudicial attitudes. It might be appropriate to further explore these aspects on a larger population, eventually using quantitative research methods. In terms of practical interventions in education contexts, we think it is important to move beyond the “culturalist” approach to relations with immigrant families, considered as merely “representatives” of their communities of origin. Rather, it is essential to bear in mind that parents have skills, experiential know-how, different styles of relationship, and strategies that have been developed as a product of the way in which families respond to the conditions they live in and have to deal with. These conditions must be improved if we want to achieve effective family-school relations that truly support the new generations of tomorrow.
